# Severe fever with thrombocytopenia syndrome virus inhibits exogenous Type I IFN signaling pathway through its NSs *in* *vitro*

**DOI:** 10.1371/journal.pone.0172744

**Published:** 2017-02-24

**Authors:** Xu Chen, Haiyan Ye, Shilin Li, Baihai Jiao, Jianqin Wu, Peibin Zeng, Limin Chen

**Affiliations:** 1 Institute of Blood Transfusion, Chinese Academic of Medical Sciences and Peking Union Medical College, Chengdu, China; 2 West China School of Public Health, Sichuan University, Chengdu, Sichuan, China; 3 Toronto General Research Institute, University of Toronto, Toronto, Ontario, Canada; Harvard Medical School, UNITED STATES

## Abstract

Severe fever with thrombocytopenia syndrome (SFTS) is an emerging infectious disease caused by a novel *bunyavirus* (SFTS virus, SFTSV). At present there is still no specific antiviral treatment for SFTSV; To understand which cells support SFTSV life cycle and whether SFTSV infection activates host innate immunity, four different cell lines (Vero, Hela, Huh7.5.1, and Huh7.0) were infected with SFTSV. Intracellular/extracellular viral RNA and expression of IFNα, and IFNß were detected by real-time RT- PCR following infection. To confirm the role of non-structural protein (NSs) of SFTSV in exogenous IFNα-induced Jak/STAT signaling, p-STAT1 (Western Blot), ISRE activity (Luciferase assay) and ISG expression (real-time PCR) were examined following IFNα stimulation in the presence or absence of over-expression of NSs in Hela cells. Our study showed that all the four cell lines supported SFTSV life cycle and SFTSV activated host innate immunity to produce type I IFNs in Hela cells but not in Huh7.0, Huh7.5.1 or Vero cells. NSs inhibited exogenous IFNα-induced Jak/STAT signaling as shown by decreased p-STAT1 level, suppressed ISRE activity and down-regulated ISG expression. Suppression of the exogenous Type I IFN-induced Jak/STAT signaling by NSs might be one of the mechanisms of SFTSV to evade host immune surveillance.

## Introduction

Severe fever with thrombocytopenia syndrome (SFTS) is an emerging infectious disease caused by a novel *bunyavirus* (SFTSV) firstly identified in China with initial mortality rates ranging from 6.3% to 30%[1–3]. Clinical manifestation of SFTSV infection includes severe fever, thrombocytopenia and leukocytopenia, and the disease process consists of four stages: incubation, severe fever, multiple organ failure, and convalescence[4, 5]. As a tick-borne disease, SFTS mainly occurred between April and October, and peaked in May to July[6]. Most of patients were farmers in endemic areas due to high risk of exposures to tick-biting[6]. Currently, ribavirin was commonly prescribed for anti- SFTSV treatment with sub-optimal efficacy[7, 8].

As a single-stranded, negative sense RNA virus, the genome of SFTSV is composed of three segments: Large (L), Medium (M), and Small (S)[1, 9, 10]. L segment consists of 6368 bp encoding RNA-dependent RNA polymerase (RdRp) which is involved in viral replication; The M segment consisting of 3378 bp has two ORFs encoding Gn and Gc, which may be a sole target for neutralizing antibodies; With length of 1744 nucleotides, S segment encodes nucleocapsid protein(NP) and non-structural protein (NSs) with 882 bp in length[1, 9, 10]. NSs was reported to have diverse functions related to the activity of viral polymerase, suppression of viral replication and blocking of interferon (IFN) production through various mechanisms in the family of Bunyaviridae[11–13]. NSs of SFTSV was described to be involved in viral replication and modulation of host response, suppressing the NF-κB pathway to facilitate SFTSV replication in human monocytic cells[14]. NSs of SFTSV was also proved to be associated with promoting viral replication through the formation of viroplasm-like structures(VLS)[15]. Many researchers had found that NSs of different viruses played important role in blocking interferon-mediated antiviral effect. One report found that non-structure protein 5 (NS5) of Dengue virus (DENV) interacted with STAT2 to block IFN signaling pathway[16]. Another research claimed that NS5A protein of Hepatitis C Virus (HCV) could disrupt STAT1 phosphorylation and suppressed Type I interferon signaling[17]. NS3/4A protein encoded by HCV genome cleaves the critical sensor molecules of the TLR3 and RIG-I signaling pathways to block the induction of type I IFNs, therefore to facilitate the establishment of a persistent infection [18]. And many viruses develop a series of mechanisms to evade the host immune response.VP24 protein of Ebola virus can interact with the STAT1 nuclear localization signal receptor, karyopherinα1, and inhibits the interaction between VP24 and PY-STAT1 to counteract with the antiviral effects of IFNβ[19, 20]. VP24 can also interact with STAT1 directly to block IFN signaling[21]. Dengue virus is capable of subverting the human IFN response by down-regulating STAT2 expression and NS4B encoded by the viral genome appeared to inhibit both IFNα/β and IFN-γ signal transduction pathways in monkey cells[22, 23]. Newcastle disease virus (NDV) V protein has been considered as an effector for IFN antagonism through suppressed STAT1 phosphorylation [24]. A more recent study demonstrated that human metapneumovirus small hydrophobic (SH) protein could inhibit STAT1 phosphorylation to block the signaling[24, 25]. A more recent study demonstrated that NSs of SFTSV could hijack STAT2 into inclusion bodies and inhibited the phosphorylation of STAT2,ultimately blocked Type I IFN signaling, but NSs had no influence on Y701 phosphorylation of STAT1[26]. Another report also found that NSs of SFTSV could reduce the phosphorylation of STAT1 at S727 but not at Y701 and inhibit Type I and Type III IFN signaling[27]. In our study, we confirmed that NSs could inhibit STAT1 phosphorylation leading to the suppression of Jak/STAT signaling.

Host innate immune response, characterized by the induction of type I IFNs (IFNα and IFNß in particular) and the activation of NK cells, is the first line of defense against various pathogen infections. Toll-like receptors (TLRs) and retinoic acid-inducible gene 1 protein (RIG-I) play an important role in this antiviral IFN response[28–30]. Following the induction of type I IFNs, the Janus kinase–signal transducer and activator of transcription (Jak/STAT) signaling pathway is activated through increased phosphorylation of STAT1 (p-STAT1) level and interferon stimulated response element (ISRE) activity to produce several hundred interferon stimulated genes (ISGs), thereby establishing an antiviral state in the cells to limit virus replication/spread[31–33]. In this study, we used Vero cells for SFTSV stock as Vero cells were defective in the production of Type I IFN due to the gene for interferon synthesis was defective or absent [34, 35]. And we demonstrated that SFTSV could activate the host innate immune responses as shown by the fact that it induces IFNα and IFNß production in Hela cells. Although SFTSV replicates in Huh7.0 and Huh7.5.1 cells efficiently, no Type I IFNs were induced in both cells possibly due to the fact that both Huh7.0 and Huh7.5.1 cells are TLR3 deficient [36–39]. In addition, we confirmed that SFTSV inhibited exogenous Type I IFN signaling pathway through NSs in Hela cells.

## Materials and methods

### Cell culture and virus

Human cervical cancer cell line (Hela) was routinely maintained in our lab. Human hepatoma cell lines Hun7.0 and Huh7.5.1 cell lines were generously provided by Dr. Ian McGilvray (University of Toronto, Canada). Hela cells were cultured in Dulbecco's modified Eagle's medium(DMEM) supplemented with 10% fetal bovine serum(FBS) and 100μg/ml penicillin and 100 μg/ml streptomycin sulfate at 37°C in 5% CO_2_ incubator. Human liver cancer cell lines Huh7.0 and Huh7.5.1 were cultured in DMEM supplemented with 10% fetal bovine serum, 100μg/ml penicillin and 100 μg/ml streptomycin sulfate,1% nonessential amino acid.

SFTSV isolated from the plasma sample of a SFTS patient in Xinyang 154Military hospital was propagated in Vero cells. Viral RNA from supernatants was extracted with a special RNA extraction Kit (Qiagen, German). The viral RNA equivalents (copy/ml)of SFTSV was measured by a reverse transcription (RT) real-time quantitative -PCR Kit for SFTSV RNA (Da An Gene Inc., Guangzhou., China) with 5μL RNA with the condition:50°C, 15min; 95°C, 15min; 94°C, 15s and 55°C, 45s for 40 cycles. The PCR amplification cycle numbers (Cts) were measured and converted into SFTSV RNA equivalents (copy/ml) using SFTSV RNA standards (recombinant pseudotyped virus, copy/ml). The supernatants from Vero cells infected by SFTSV were collected at 72 hours after inoculation. Hela, Huh7.0, Huh7.5.1 cells and Vero cells were infected by supernatants mentioned above at 48 hours before harvesting the cells and supernatants for further detections.

### Construction of NSs over-expression plasmid

Open reading frame(ORF) encoding NSs was amplified by reverse transcription-PCR(RT-PCR) from SFTSV genomic RNA, and two restriction enzymes Xhol and PremI (New England Biolabs, USA) were used to cloned the NSs segment into expression vector pc-DNA3.1 with three tag (Flag, His, StrepII) using T4 ligase (Toyobo, Japan). The primers for SFTSV NSs plasmid construction were: forward: 5’-TTAGACCTCCTTCGGGAGGTCA-3’; reverse: 5’-TGGAGCATTTGCTCAG CGACAT-3’.

### Detection of ISRE activity using ISRE-luciferase reporter gene assay

ISRE activity is an indicator of activation of Type I IFN signaling. In this study, Hela cells cultured in 24-well plates were co-transfected with pISRE-luc reporter plasmid(4μg/ml), pRL –TK plasmid(4ng/ml),and indicated amount of expression plasmid of NSs (2μg/ml) by 2μL polyethylenimine(PEI) per well. 24 hours post-transfection, cells were stimulated with100IU/ml IFN-α for 24hours, and then Hela cells were collected with100μL 1× passive lysis buffer(PLB) in each well, then the relative luciferase activity was measured with a dual-luciferase reporter (DLR) assay kit (Promega, USA) following manufacturer’s protocol.

Similarly, to observe the influence of SFTSV on the activation of ISRE, Hela cells were co-transfected with the pISRE-luc reporter plasmid and pRL–TK plasmid for 12 hours before the cells were infected with SFTSV (MOI = 1.0) for 24hours, after that, cells were stimulated with 100IU/ml IFN-α for 24hours before cells were collected for dual-luciferase reporter gene assay as above.

### Detection of the expressions of IFNα, IFNß, TLR3, RIG-I and ISGs at mRNA level

Total RNA was isolated from Hela cells using a total RNA purification kit (Invitrogen,USA). Reverse transcription (RT) of the mRNA was carried out by First Strand cDNA Synthesis Kit (Bio-rad, USA) using random primers with conditions of 25°C for 5min; 42°C for 30min and 85°C for 5min.The real-time PCR was performed with the FastStart Universal SYBR Green Master Mix (Roche, USA). The 19μL reaction mixture with 1μL cDNA was first denatured at 95°C for 5 min and then 40 cycles of PCR were performed using the following protocol: 95°C 30 s; 60°C 30 s; 72°C 30 s in 20μl reaction volume. mRNA levels of IFNα, IFNß, TLR3, RIG-I and ISGs including: IFN-induced ubiquitin-like protein (ISG15), myxovirus resistance A (MxA), 2’,5’-oligoadenylatesynthetase3 (OAS3) were determined by real-time quantitative PCR. The housekeeping geneglyceraldehydes-3-phosphate dehydrogenase (GAPDH) was chosen as reference gene to quantify the level of mRNA expression. The primers used for real-time quantitative PCR in this study were listed in Table 1.

**Table 1:** The list of primers for RT real-time qualitative PCR to detect the expression of IFNα, IFNß, TLR3, RIG-I and ISGs.

Gene	Forward primer	Reverse primer
**IFNα**	5′-TCGCCCTTTGCTTTACTGAT-3′	5′-GGGTCTCAGGGAGATCACAG-3′
**IFNβ**	5′-AAACTCATAGCAGTCTGCA-3′	5′-AGGAGATCTTCAGTTTCGGAGG-3′
**RIG-I**	5'-CTTGGCATGTTACACAGCTGAC-3'	5'-GCTTGGGATGTGGTCTACTCA-3'
**TLR3**	5'-TCCCAAGCCTTCAACGACTG-3'	5'TGGTGAAGGAGAGCTATCCACA-3′
**ISG15**	5'-CGCAGATCACCCAGAAGATT-3'	5'-GCCCTTGTTATTCCTCACCA-3'
**MxA**	5'-GTGCATTGCAGAAGGTCAGA-3'	5'-CTGGTGATAGGCCATCAGGT-3'
**OAS3**	5'-GTCAAACCCAAGCCACAAGT-3'	5'-GGGCGAATGTTCACAAAGTT-3'
**GAPDH**	5'-GCCTCCTGCACCACCAACTG-3'	5'-ACGCCTGCTTCACCACCTTC-3'

### Protein isolation and western blot analysis

Hela cells in 6-well plates were washed three times with PBS and then lysed by radio-immune precipitation assay (RIPA) with lysis buffer(1% Triton X-100, 1% deoxycholate, 0.1% SDS) (Beyotime, China).The protein concentration was quantified by BCA Protein Assay Kit (Beyotime,China) and the total proteins were subjected to SDS-PAGE and then transferred to a polyvinylidenefluoride (PVDF) membrane (Millipore, USA). After blocking with 5% skim milk or 5% Bovine serum Albumin (BSA) in TTBS buffer (1L TTBS contains 8.77g NaCl, 2.42g Tris, 1g Tween, pH7.5) for 2 hours, the membrane was incubated with primary antibodies overnight at 4°C before it was washed with 1xTTBS for 3 times, then it was incubated with horseradish peroxidase-conjugated secondary antibodies for 1 hour followed by wash with 1xTTBS for 3 times, 10 minutes each. Protein bands were detected by enhanced chemiluminescence (ECL) kit (Millipore, USA) and visualized by LAS4000 mini (GE, USA). The primary antibodies used in this study including: mouse anti-flag antibody, mouse anti-GAPDH antibody and mouse anti-β-Actin (Abbkine, Redlands, CA), rabbit anti-STAT1, rabbit anti-Phospho-STAT1 (Y701) (Cell Signaling Technology, Inc., Beverly, MA). The secondary antibodies were HRP-conjugated ECL goat anti-rabbit IgG, or HRP-conjugated ECL sheep anti-mouse IgG (Abmart, China).

### Enzyme-Linked Immunosorbent Assay(ELISA) for IFNα and IFNβ

Hela cells and Vero cells were infected with SFTSV (MOI = 1.0) or untreated (only 500μL medium) for 2 hours in 24-plate well and cultured for 48 hours respectively. The culture supernatant was harvested and assayed for IFNα and IFNβ with Human Interferonα ELISA Kit and Human Interferonβ ELISA Kit (Abmart, China) following the manufacturer’s protocol. The expression levels of IFNα (pg/ml), IFNβ (ng/ml) protein in culture medium stimulated by SFTSV in Hela cells and Vero cells were calculated based on a standard curve.

### Statistical analysis

A two-tailed Student^’^s t test or one-way ANOVA were used to evaluate the data and p<0.05 was considered statistically significant. All experiments were repeated at least three times.

## Results

### SFTSV replicates in Vero, Hela, Huh7.0 and Huh7.5.1 cells efficiently

**Fig 1:**
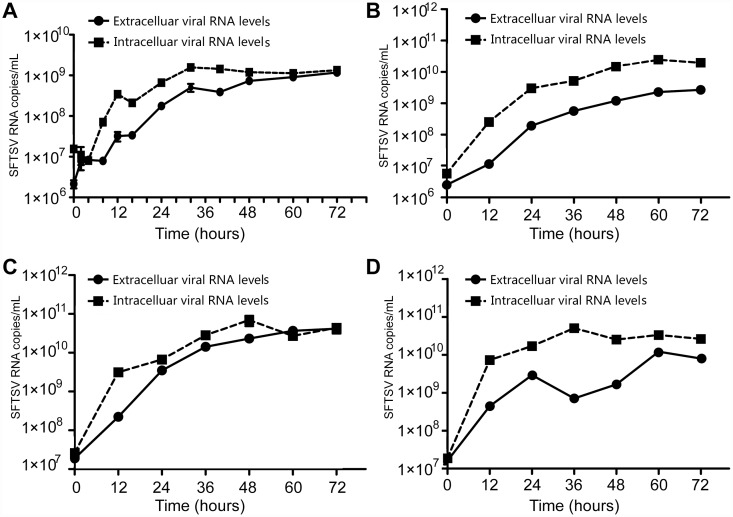
SFTSV replicates in Vero, Hela, Huh7.0, Huh7.5.1 cells efficiently. SFTSV infected Vero (A), Hela (B), Huh7.0 (C) and Huh7.5.1 (D) cells at MOI = 0.09. Cells and culture supernatant were collected at different time-points. Viral RNA levels was measured by Fluorescence Quantitative Polymerase Chain Reaction Diagnostic Kit as described in material and methods. Data were presented as means± SD, n = 3. Data were representative of at least 3 repeated experiments.

We infected Vero, Hela, Huh7.0 and Huh7.5.1 cells with SFTSV (MOI = 0.09) or uninfected in 24-well plate, and collected the cells and supernatant at different time points. RNA levels were detected by a real-time quantitative RT-PCR Kit as described in materials and methods. As shown in Fig 1, SFTSV replicated in Vero, Hela, Huh7.0, and Hun7.5.1 cells efficiently. In all these cells, the intracellular viral RNA levels were always higher than that secreted into corresponding culture medium. The viral RNA levels reached its maximum 72hrs post infection[Fig 1].

### SFTSV activates host innate immunity in Hela cells but not in Vero, Huh7.0 or Huh7.5.1 cells

**Fig 2:**
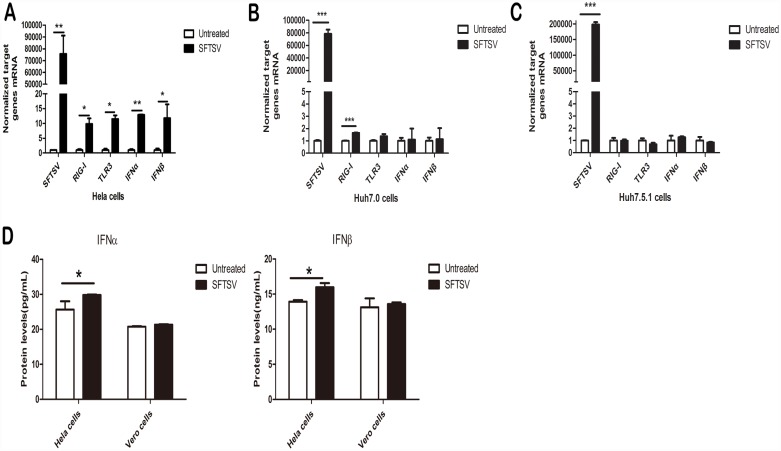
SFTSV activates host innate immunity in Hela cells. Endogenous IFNα/β were up-regulated by SFTSV in Hela cells but not in Huh7.0 0r Huh7.5.1 cells. Hela(A), Huh7.0(B) and Huh7.5.1(C) cells were infected with1.0 MOI SFTSV for 2 hours and cultured for 48 hours before RNA were collected with Trizol reagent following the manufacturer’s protocol. The levels of TLR3, RIG-I, IFNα, IFNβ, GAPDH mRNA and SFTSV mRNA were measured by quantitative RT-PCR. (D) Protein levels of IFNα/β were up-regulated in the culture medium of Hela cells stimulated by SFTSV. Hela and Vero cells were infected with SFTSV (MOI = 1.0) for 2 hours and cultured for 48 hours before the culture medium were collected, The protein levels of IFNα/β were assayed by the Elisa Kit. Data were presented as mean± SD (n = 3). *p<0.05,**p<0.01, ***p<0.001.Data are representative of at least 3 repeated experiments.

Following successful infection of SFTSV in Hela cells, both IFNα and IFNβ were induced properly in the late stage following SFTSV infection (Fig 2A). In the meantime, as the two major sensor molecules in innate immune response, RIG-I and TLR3 mRNA expression levels were also increased. Simultaneously, human liver cancer cell lines Huh7.0 (TLR3 -/-) and Huh7.5.1 (TLR-/- RIG-I -/-) were infected with SFTSV (MOI = 1.0) for 2 hours and cultured for 48hours, expression RNA levels of IFNα and IFNβ were quantified by real-time PCR. As shown in Fig 2A, 2B and 2C, both IFNα and IFNβ were not induced following SFTSV infection in either Huh7.0 or Huh7.5.1 cells. Simultaneously, the protein levels of IFNα and IFNβ in culture medium of Hela cells and Vero cells were determined by ELISA, and we found that IFNα and IFNβ were up-regulated in the culture medium of Hela cells but not in Vero cells stimulated by SFTSV as shown in Fig 2D, These results collectively demonstrated that SFTSV may activate host innate immunity through TLR3 signaling pathway.

### SFTSV inhibits exogenous IFNα-induced Jak/STAT signaling pathway

**Fig 3:**
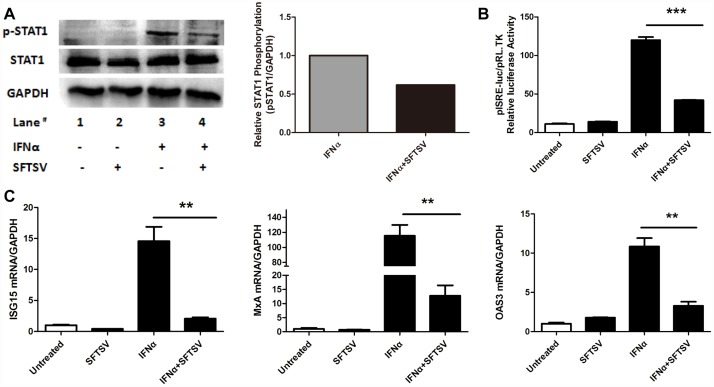
SFTSV inhibits exogenous Type I IFN signaling pathway. (A)**SFTSV inhibits the phosphorylation of STAT1**. Hela cells were infected with SFTSV(MOI = 1.0) for2 hours and cultured for 36 hours before 100IU/ml IFNα was added for 15 minutes,and total protein were collected for WB and densitometry analysis for p-STAT1. (B)**SFTSV suppresses ISRE activity.** Hela cells were co-transfected with ISRE-luc plasmid 2μg per well and pRL-TK plasmid 2ng per well, then Hela cells were infected with SFTSV (MOI = 1.0) for 2 hours and cultured for 24hours, then we used 100IU/ml IFNα to stimulated the Hela cells for 24 hours, and samples were collected for dual-luciferase reporter assay. (C)**SFTSV down –regulates the expression of ISGs.** Hela cells were infected with 1.0MOI SFTSV for2 hours and culturedfor24hours, then treated with 100IU/ml IFNα for 12 hours, and total RNAs were isolated with Trizolreagent, The levels of ISG15, MxA, OAS3, GAPDH mRNA were measured by quantitative RT-PCR. Data were presented as mean± SD, n = 3. **p<0.01, ***p<0.001. Data are representative of at least 3 repeated experiments.

To confirm the effect of SFTSV infection on the exogenous IFNα-induced Jak/STAT signaling, Hela cells were infected with SFTSV for 2 hours and cultured for 36hours before IFNα was added to stimulate the cells for 15min, and total proteins were collected for detection of the phosphorylated STAT1.As shown in Fig 3A, TheY701 phosphorylation level of STAT1 was inhibited by SFTSV infection. At the same time, we examined IFN-induced ISRE activity by dual-luciferase reporter(DLR) gene assays. Hela cells were co-transfected with ISRE-luc plasmid and pRL-TK plasmid for 12 hours before they were infected with SFTSV for 2 hours and cultured for 24hours, then IFNα was added to the cells for 24 hours. As shown in Fig 3B, SFTSV inhibited the IFNα-induced ISRE activity. Further more, we tested the expression levels of several IFN-induced ISGs, such as ISG15, MxA, and OAS3 by real-time quantitative PCR. As expected, SFTSV inhibited ISG expression [Fig 3C]. These data collectively confirm that SFTSV infection can inhibit exogenous IFNα-induced Jak/STAT signaling at 3 different levels: decreased p-STAT1, suppressed ISRE activity and down-regulated ISG expression.

### SFTSV inhibits exogenous IFNα-induced Jak/STAT signaling pathway through NSs

**Fig 4:**
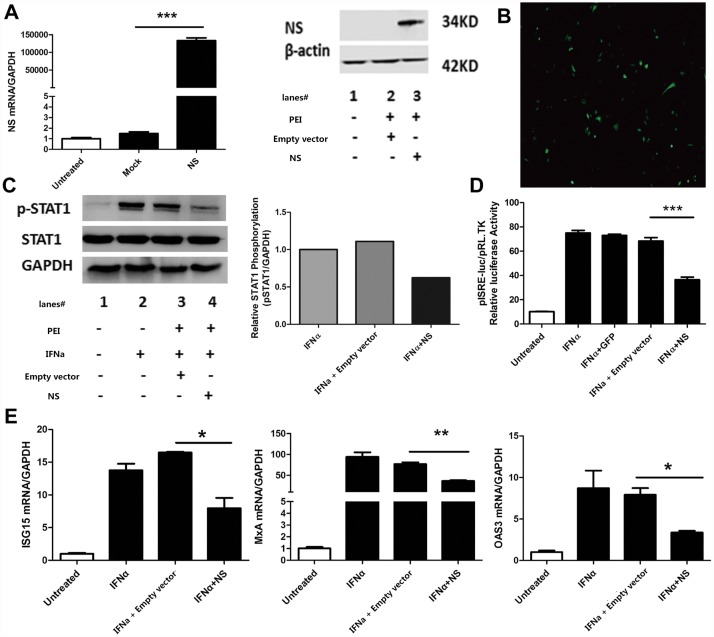
SFTSV inhibits exogenous IFNα-induced Jak/STAT signaling pathway through NSs. (A)**NSs of SFTSV expresses in Hela cells successfully.** Hela cells were transfected with empty vector pcDNA3.1 plasmid or NSs plasmid for 48hours, and total RNA were prepared with Trizol reagent, cDNA were harvested with reverse transcriptase for real time quantitative PCR. Protein samples were harvested for WB analyzed. (B) **Expression of GFP protein in Hela cells.** GFP-expressing plasmid DNA was transfected into Hela cells for 48 hours and GFP expression was observed under immunofluorescence microscope (200×). (C)**NSs inhibits the phosphorylation of STAT1.**Hela cells were transfected with NSs plasmid or empty vector pcDNA3.1 for 36 hours before IFNα was added to stimulate the cells for 15minutes. Total proteins were collected with protein lysis buffer for WB and densitometry analysis of western blot datafor p-STAT1. (D)**NSs suppresses the ISRE activity**. Hela cells were co-transfected with pISRE-luc plasmid and pRL-TK plasmid together with NSs plasmid, GFP-expressing plasmid or empty vector pcDNA3.1 plasmid for 24hours, and IFNα (100IU/mL)were added to the cells for 24hours, then samples were collected for dual-luciferase reporter assay. (E) **NSs down-regulates the expression of several ISGs.** Hela cells were transfectd with NSs plasmid or empty vectorpcDNA3.1 plasmid for 36hours, then we added IFNα (100IU/ml) to the cells for 12hours,RNA were collected with Trizol reagent, cDNA were used for real time quantitative PCR. Data were presented as mean± SD, n = 3.*p<0.05, **p<0.01, ***p<0.001.Data were representative of at least 3 repeated experiments.

Previous studies have demonstrated that NSs of SFTSV could block Type I and Type III signaling through inhibition of the phosphorylation level of STAT1 (S727) and STAT2 (Y690)[26, 27]. To confirm the role of NSs in IFN signaling, we first constructed the NSs over-expression plasmid and used GFP-expressing plasmid as control. As shown in Fig 4A, NSs gene was successfully over-expressed both at mRNA and at protein level in Hela cells. GFP protein was expressed successfully in Hela cells as green fluorescence was observed under immunofluorescence microscope as shown in Fig 4B. More interestingly, NSs inhibited exogenous IFNα-induced Jak/STAT signaling as shown by the decreased Y701 p-STAT1 (Fig 4C), suppressed ISRE activity (Fig 4D), and down-regulated ISG expression (Fig 4E). As shown in Fig 4D, we found that GFP protein had no influence on the activity of ISRE, ruling out the possibility that the effects observed was simply due to the over expression of any protein. These data confirm that SFTSV inhibits exogenous IFNα-induced Jak/STAT signaling pathway through NSs.

## Discussion

Pathogen-associate molecule patterns (PAMPs) present on the surface or genome of the pathogens activate various down-stream signaling pathways to induce type I IFN production[33]. TLR3 plays an important role in IFN-mediated innate immunity against many virus infections. One of the examples is that HSV-2 activates TLR3 siganling and inhibits its replication[40]. In this study, we demonstrated that SFTSV infected Vero, Hela, Huh7.0, and Huh7.5.1 cells efficiently and SFTSV RNA could be detected in these four cells as shown in Fig 1. Both IFNα and IFNβ were successfully induced following SFTSV infection in Hela cells, but not in Huh7.0, Huh7.5.1 cells or in the culture medium of Vero cells (Fig 2). It has been well-known that TLR3 is deficient in both Huh7 and Huh7.5.1 cells, and Vero cells are also defective in IFN production. Our data indicated that SFTSV activated host innate immunity possibly through TLR3 signaling. However, the detailed molecular mechanism remains further studies, including TLR3 knock-in in Huh7.0 and Huh7.5.1 cells.

Following the induction of type I IFNs, IFNα and/or IFNβ binds to the same receptor (IFNAR) to activate the traditional Jak/STAT signaling pathway, leading to the production of several hundred ISGs to establish an anti-viral state within cells to limit virus replication or spread. Many of these ISGs have been identified as anti-viral proteins, such as MxA, OAS3, ISG15[33]. However, recent studies from HCV alluded that the anti-viral activity of some ISGs may be virus specific. Chen, et al demonstrated that ISG15 and ISG16 promoted HCV production and blunted IFNα anti-viral activity[41, 42]. ISG12a can inhibit HCV replication through activation of the JAK/STAT signaling pathway[43]. A recent study has found that ISG15 conjugation can stimulate Hepatitis B Virus(HBV)production independent of Type I IFN signaling pathway[44]. All these data point out virus-host interaction is of tremendous importance in the battle against virus infection. In our study, we first showed that SFTSV inhibited exogenous IFNα-induced Jak/STAT signaling through decreasing the level of Y701 p-STAT1, suppressing ISRE activity and down-regulating ISGs expression. Then we confirmed that this inhibition was mediated through NSs of SFTSV in Hela cells, which is contradictory to previous studies that NSs decreased S727 p-STAT1 in HEK293 cells[27], which may result from the different cell lines in respective experiments, but the detailed mechanism need to be further studied. However, our result is complementary to the most recent studies showing that NSs of SFTSV inhibits host innate immunity through suppression of both type I and type III IFN signaling[26, 27].

In summary, our current data support that SFTSV activates endogenous Type I IFN in Hela cells and SFTSV inhibits exogenous IFNα-induced Jak/STAT signaling through its encoded NSs. Our data provided evidence that close interaction of SFTSV with host innate immune system exists, which offered better understanding of the viral pathogenesis.
